# Integration of Machine Learning and Experimental Validation to Identify Anoikis-Related Prognostic Signature for Predicting the Breast Cancer Tumor Microenvironment and Treatment Response

**DOI:** 10.3390/genes15111458

**Published:** 2024-11-12

**Authors:** Longpeng Li, Longhui Li, Yaxin Wang, Baoai Wu, Yue Guan, Yinghua Chen, Jinfeng Zhao

**Affiliations:** 1 Institute of Physical Education and Sport, Shanxi University, Taiyuan 030006, China; 202223702005@email.sxu.edu.cn (L.L.);; 2 School of Kinesiology and Health, Capital University of Physical Education and Sports, Beijing 100191, China

**Keywords:** breast cancer, anoikis, machine learning, tumor microenvironment, prognostic signature

## Abstract

**Background/Objectives:** Anoikis-related genes (ANRGs) are crucial in the invasion and metastasis of breast cancer (BC). The underlying role of ANRGs in the prognosis of breast cancer patients warrants further study. **Methods:** The anoikis-related prognostic signature (ANRS) was generated using a variety of machine learning methods, and the correlation between the ANRS and the tumor microenvironment (TME), drug sensitivity, and immunotherapy was investigated. Moreover, single-cell analysis and spatial transcriptome studies were conducted to investigate the expression of prognostic ANRGs across various cell types. Finally, the expression of ANRGs was verified by RT-PCR and Western blot analysis (WB), and the expression level of PLK1 in the blood was measured by the enzyme-linked immunosorbent assay (ELISA). **Results:** The ANRS, consisting of five ANRGs, was established. BC patients within the high-ANRS group exhibited poorer prognoses, characterized by elevated levels of immune suppression and stromal scores. The low-ANRS group had a better response to chemotherapy and immunotherapy. Single-cell analysis and spatial transcriptomics revealed variations in ANRGs across cells. The results of RT-PCR and WB were consistent with the differential expression analyses from databases. NU.1025 and imatinib were identified as potential inhibitors for SPIB and PLK1, respectively. Additionally, findings from ELISA demonstrated increased expression levels of PLK1 in the blood of BC patients. **Conclusions:** The ANRS can act as an independent prognostic indicator for BC patients, providing significant guidance for the implementation of chemotherapy and immunotherapy in these patients. Additionally, PLK1 has emerged as a potential blood-based diagnostic marker for breast cancer patients.

## 1. Introduction

Breast cancer, a leading cause of morbidity among women, has overtaken lung cancer as the most commonly diagnosed cancer type in women [1]. According to the American Cancer Society’s 2023 report, there were over 290,000 new cases in the United States alone, accounting for roughly one-third of all female cancer diagnoses [2]. With the development of multiple treatment options in recent years, 70–80 percent of breast cancer patients are considered to be curable [3]. Despite dramatic advances in survival rates over the last two decades, the disease’s global prevalence continues to climb [4]. Therefore, seeking new and accurate prognostic markers is crucial for predicting the prognosis of breast cancer patients.

Cell death is an integral part of the growth and maintenance of organisms, categorized into programmed cell death (apoptosis and autophagy) and non-programmed cell death (necrosis) [5]. Programmed cell death is a highly regulated process designed to remove unwanted or damaged cells, whereas non-programmed death is usually triggered by pathological injury [6]. In breast cancer, disruption of cell death-related mechanisms often leads to aberrant proliferation of tumor cells and evasion of immune surveillance. For example, inhibition of apoptosis and dysregulation of autophagy are closely associated with cancer aggressiveness and treatment resistance [7,8]. In addition, it has been found that there is a mutual crosstalk between autophagy and apoptosis, together influencing the progression of breast cancer [9,10]. Given the role of cell death in breast cancer, building the cell death-related prognostic model will provide a new idea for the development of precision medicine in breast cancer.

Anoikis is a programmed form of cell death that occurs when cells detach from the extracellular matrix or lose interactions with neighboring cells, leading to apoptosis [11]. This key mechanism prevents abnormal cell growth and attachment to improper extracellular matrices [12,13]. However, cancer cells often develop resistance to anoikis, enabling them to survive and proliferate independently after detaching from the primary tumor site [14]. ANRGs play a crucial role in tumor invasion and metastasis [15]. For instance, TCF7L2 promotes gastric cancer cell proliferation, anoikis resistance, and migration through a PLAUR-dependent pathway [16]. Additionally, inhibiting *BMP4* gene expression can reduce anoikis resistance in breast cancer cells, offering a new perspective for preventing breast cancer invasion and metastasis [17]. Moreover, transforming growth factor β receptor 3 (TGFBR3) has been identified as a regulator of anoikis, and its inhibition decreases breast cancer cells’ sensitivity to anoikis [18]. However, despite these promising findings, the use of ANRGs as prognostic markers in breast cancer remains challenging. Influenced by various signaling pathways and the tumor microenvironment, it makes the identification of reliable biomarkers more complex. In addition, clinical validation of ANRGs as a prognostic tool in breast cancer remains limited, and their predictive performance remains unsatisfactory. Therefore, using more cutting-edge methods (machine learning and single-cell analysis) to further investigate the specific roles of ANRGs and their interactions with the tumor microenvironment is crucial to understanding their true potential as prognostic markers in breast cancer.

In this study, we employed machine learning to identify prognostic ANRGs, which could serve as potential therapeutic targets for breast cancer. Subsequently, we constructed the ANRS based on these genes and explored the potential clinical applications of ANRGs and ANRS. We also explored the relationship between the ANRS and various factors, including the tumor microenvironment, chemotherapy drug sensitivity, and immunotherapy efficacy. These findings provide new insights for the clinical monitoring and treatment of breast cancer.

## 2. Materials and Methods

### 2.1. Data Acquisition and Processing

ANRGs were sourced from the GeneCards database (https://www.genecards.org/, accessed on 24 February 2024) (Table S1). Gene expression data and clinical data for TCGA-BRCA were retrieved from The Cancer Genome Atlas (TCGA) database (https://portal.gdc.cancer.gov/, accessed on 25 February 2024). Similarly, gene expression and clinical data for METABRIC were obtained from the cBioPortal for Cancer Genomics (https://www.cbioportal.org/, accessed on 27 February 2024). Gene expression data and clinical data for GSE96058, GSE86166, GSE45827, GSE57297, and GSE24124 were downloaded from the Gene Expression Omnibus (GEO, https://www.ncbi.nlm.nih.gov/geo/, accessed on 28 March 2024). Genes were annotated according to the platform corresponding to each dataset, and detailed information on the GEO data is provided in Table S2. The TCGA dataset was downloaded with the TPM data, and the count data from the GEO datasets was converted to TPM data. In the gene expression data, genes whose expression was not equal to 0 in 70% of the samples were extracted. Samples with a survival time of 0 and missing clinical characteristics were removed from the clinical data. Basic information about the clinical data is detailed in Table S3. Differential analysis of the genes in TCGA-BRCA was performed using the “easyTCGA” R package (version 0.0.1.7000). The overlap between differentially expressed genes (DEGs) and ANRGs was then identified for subsequent analysis.

### 2.2. Screening of the Prognostic ANRGs

Firstly, one-way Cox regression analysis was performed on the intersecting genes, and the prognostic ANRGs with *p* < 0.05 were extracted. Subsequently, six machine learning algorithms were applied to refine the selection of key ANRGs. The Lasso and Enet algorithms were implemented using the glmnet package [19], with lambda.min selected as the parameter, and the genes with coef not equal to 0 were extracted as the core genes. The RSF algorithm was executed through the randomForestSRC package [20], with the parameter nodesize obtained through the tune.nodesize function, and the parameter ntree set to 1000. The Coxboost algorithm was executed through the CoxBoost package [21], and the core genes were obtained through the model constructed by the optimal step. The importance of the genes in the GBM model was analyzed through the gbm.perf function of the gbm package [22], and the top 10 most important ones were selected as core genes. The importance of genes in the XGBoost model was analyzed by the xgb.importance function of the XGBoost package [23], and the top 10 most important ones were selected as core genes. Independent key ANRGs were further screened by multifactor Cox.

### 2.3. Construction of the ANRS

For this study, the TCGA-BRCA and METABRIC cohorts were designated as the training sets, while GSE96058 and GSE86166 served as the validation sets. Referring to the method in the previous study [24], we chose the machine learning algorithm with the highest average C index in both cohorts to construct the ANRS. The 14 machine learning algorithms are Lasso, Enet, Ridge, Survival-Svm, GBM, SuperPC, CoxBoost, StepCox, plsRcox, SurvReg, CoxPH, ObliqueRSF, GlmBoost, and Rpart. The Lasso, Ridge, and Enet algorithms were implemented using the glmnet package, with the regularization parameter lambda determined through 10-fold cross-validation. Lasso was applied when α was set to 1, Ridge when α was 0, and Enet was used for other α values. The Survival-Svm algorithm was executed with the “survivalsvm” function from the survivalsvm package (version 0.0.5). The GBM and SuperPC algorithms were fitted using 10-fold cross-validation via the gbm and SuperPC packages [25], respectively. For CoxBoost, we first optimized the penalty term using the “optimCoxBoostPenalty” function, followed by 10-fold cross-validation to determine the best boosting steps, with final fitting achieved using the “CoxBoost” function. The stepwise Cox model was constructed with the survival package (version 3.5-5), while the plsRcox model was built using the plsRcox package [26]. The SurvReg algorithm was constructed using the “survreg” function of the survival package. The CoxPH algorithm was constructed using the “coxph” function of the survival package. The ObliqueRSF algorithm was implemented through the “ORSF” function of the obliqueRSF package (version 0.1.2). The GlmBoost algorithm was built through the mboost package (version 2.9-10). The Rpart algorithm is implemented through the Rpart package (version 4.1.23). Based on the median ANRS value, patients were stratified into low-ANRS and high-ANRS groups, with Kaplan–Meier survival analysis conducted to compare overall survival (OS) between the groups. Furthermore, ROC curves were generated for the ANRS at 1, 3, and 5 years to assess its predictive accuracy.

### 2.4. Construction of the Nomogram

The association between clinical features and the ANRS was examined using both univariate and multivariate Cox regression analyses. Following this, a nomogram incorporating clinical features and the ANRS was developed using the regplot package (version 1.1). The performance of the nomogram was evaluated by generating ROC and calibration curves using the rms package (version 6.1-0).

### 2.5. Functional Enrichment Analysis

Differentially expressed genes between the low-ANRS and high-ANRS groups were identified using the “limma” R package [27], with a threshold of log2|FC| ≥ 1 and *p* < 0.05. Gene Ontology (GO) and Kyoto Encyclopedia of Genes and Genomes (KEGG) enrichment analyses were carried out using the “clusterProfiler” R package [28]. Additionally, functional and pathway variations across different ANRS groups were assessed with the “GSVA” R package [29].

### 2.6. Immune Cell Infiltration Analysis

Immune cell infiltration in the high- and low-ANRS groups was evaluated using the CIBERSORT, EPIC, TIMER, MCPcounter, quantiseq, and ESTIMATE algorithms from the “IOBR” R package [30]. Additionally, the relationship between the ANRS and immune checkpoints was explored, and tumor microenvironment-related scores for breast cancer patients were assessed using the TIDE algorithm (http://tide.dfci.harvard.edu/, accessed on 18 April 2024).

### 2.7. Drug Sensitivity Analysis and Immunotherapy Analysis

Drug sensitivity analysis of chemotherapy-related drugs was performed using the “oncoPredict” R package [31], with half-maximal inhibition concentrations (IC50) indicating drug sensitivity. The response of breast cancer patients to immunotherapy was also evaluated. Immunophenotype score (IPS) data were downloaded from TCIA (https://tcia.at/, accessed on 19 April 2024) and analyzed for differences between different ANRS groups. IPS was determined to be a good predictor of response to anti-PD-1 and anti-CTLA-4 therapy.

### 2.8. Single-Cell Analysis and Spatial Transcriptome Analysis

The expression of ANRGs in different cell types was analyzed using the single-cell database TISCH2 [32]. Additionally, the spatial distribution of ANRGs was analyzed using the Sparkle database (https://grswsci.top/analyze, accessed on 21 April 2024).

### 2.9. Validation of the Prognostic ANRGs

The expression of ANRGs in various datasets was investigated using DiSignAtlas (http://www.inbirg.com/disignatlas/, accessed on 23 April 2024). ANRGs expression data in cell lines was obtained from the CCLE database (https://depmap.org/portal/download/all/, accessed on 24 April 2024), with visualizations illustrating ANRG expression across normal cell lines (HMEL) and breast cancer cell lines (MCF7, MDAMB453, and BT20). Immunohistochemical results for ANRGs were obtained from the HPA database (https://www.proteinatlas.org, accessed on 27 April 2024).

### 2.10. Connectivity Map (CMap) Analysis

In accordance with methodologies outlined in earlier research [33,34,35], drug characteristics were retrieved from the Connectivity Map database (https://clue.io/, accessed on 30 April 2024), with the initial 150 upregulated and 150 downregulated expression profiles chosen for input. The CMap scores were calculated using the Extreme Sum (XSum) algorithm, and the three drugs with the lowest CMap scores were selected for visualization. The lower the CMap score of a drug, the greater its potential for treating diseases.

The proteins and small-molecule drugs with the lowest CMap scores were selected for molecular docking. Protein three-dimensional structures were obtained from the Uniprot database (https://www.uniprot.org/, accessed on 10 May 2024). Three-dimensional structures for small-molecule drugs were sourced from PubChem (https://www.ncbi.nlm.nih.gov/pccompound, accessed on 13 May 2024). The process of molecular docking entailed the preparation of proteins and ligands, grid creation, and the docking of compounds. Detailed operation: 1. Import the protein structure into PyMOL software (version 2.3.1), remove water molecules, metal ions, and other ions in the structure, and then save the modified structure in the file format of pdb. 2. AutoDockTools (version 1.5.6) in the software to import the protein structure and small-molecule structure, process them, and then save them in the format of PDBQT. 3. Create the docking box. Import protein molecules and small molecules, set docking box parameters, include all protein molecules inside the box and drag ligands outside the box, then save and export as a GPF file. 4. Run AutoDockTools to calculate the GPF file saved in the previous step and then save it. 5. Set up the large-molecule file and small molecules. Set the search parameters and docking parameters and then save the DPF file. 6. Import the DPF file by running AutoDock and view the docking results. 7. Visualize the docking results in PyMOL.

### 2.11. Integrated Analysis of PLK1

We assessed the expression levels of PLK1 in tumor tissues and paired adjacent normal tissues then investigated its differential expression in relation to various clinical features. The diagnostic value of PLK1 in the TCGA-BRCA cohort was evaluated using the “tidymodels” R package (version 1.2.0), and ROC curves were plotted. The survival value of PLK1 across different datasets was assessed using Kaplan–Meier analysis, and a meta-analysis was conducted with the “meta” R package [36]. Additionally, the functional status associated with PLK1 in breast cancer was explored using CancerSEA.

### 2.12. ELISA Kit Test

Blood and tissue samples from BC patients were provided by Shanxi Cancer Hospital, with the study receiving approval from the Shanxi Cancer Hospital Research Ethics Committee (Approval No. KY2023163). All participants signed informed consent forms. The PLK1 ELISA kit was purchased from Wuhan Fein Biological Technology Co., Ltd. (Wuhan, China), and experiments were conducted according to the kit’s instructions.

### 2.13. RT-PCR

Total RNA was extracted from adjacent non-tumor and tumor tissues using Trizol reagent (Herui, Fujian, China), and reverse transcription was carried out using the Takara kit (Takara Bio, Beijing, China). Real-time quantitative PCR (RT-PCR) involved preparing the reverse transcription system, performing the PCR reactions, and calculating the mRNA expression levels of the target genes. The primers for the five ANRGs are provided in Table S4.

### 2.14. Western Blot Analysis

Cancer or paracancer tissues were homogenized by adding lysate and centrifuged at 12,000 rpm at 4 °C for 5 min, and the tissue supernatant was collected. The protein concentration in the supernatant was detected by the BCA kit (P0010, Biyun Tian, Shanghai, China). Proteins were separated by SDS-PAGE gel electrophoresis and transferred to a PVDF membrane. This was followed by incubation with a primary antibody, and the membrane was washed with TBST and incubated with a labeled secondary antibody for half an hour. The primary antibodies for this study were as follows: anti-SPIB (1:1000, CBA-13887, Cobio, Shanghai, China), anti-PLK1 (1:1000, CBA-07506, Cobio, Shanghai, China), anti-NTRK3 (1:1000, FNab09003, Fine Test, Wuhan, China), anti-EDA2R (1:1000, CBA-02375, Cobio, Shanghai, China), anti-CD24 (1:1000, CBA-03850, Cobio, Shanghai, China), and anti-GAPDH (1:10000, 60004-1-Ig, Proteintech, Wuhan, China ). The target bands were detected using a ChemiScope 6100 Chemiluminescent Imaging System (CLINX, Shanghai, China ), observed and analyzed on a gel imaging system, and photographed. The gray value of the proteins was determined by AlphaEaseFC software (version 4.0), and the ratio of the target protein to the internal reference protein was calculated to derive the relative expression of the proteins.

### 2.15. Statistical Analysis

The Wilcoxon test was applied to compare differences between two groups. Kaplan–Meier (KM) analysis was used to evaluate the differences in overall survival (OS) between the low-ANRS and high-ANRS groups [37]. All statistical analyses were performed using R version 4.3.0, with a *p*-value of less than 0.05 considered statistically significant.

## 3. Results

### 3.1. Screening for ANRGs Associated with Prognosis

**Figure 1:**
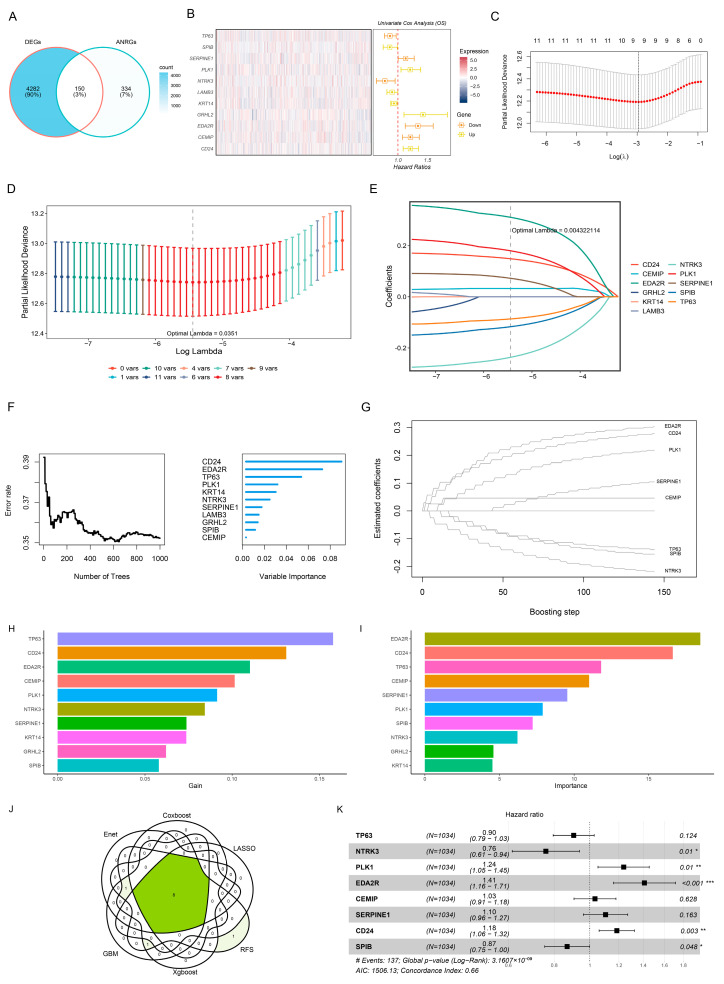
Screening of the prognostic ANRGs. (**A**) Venn diagram showing the intersection of DEGs with ANRGs. (**B**) Univariate Cox analysis of ANRGs. (**C**) Screening of the prognostic ANRGs by the Enet algorithm. (**D**) Plot of ten-fold cross-validations. (**E**) Plot of LASSO coefficient. Screening of the prognostic ANRGs by random forest algorithm (**F**) and Coxboost (**G**). (**H**) Top 10 most significant genes screened by the xgboost algorithm. (**I**) Top 10 most significant genes screened by the GBM algorithm. (**J**) Venn plot shows the intersected prognostic ANRGs identified by six machine learning algorithms for survival. (**K**) Multivariate Cox analysis of the prognostic ANRGs. * *p* < 0.05; ** *p* < 0.01; *** *p* < 0.001.

In TCGA-BRCA, a total of 4432 DEGs were identified using thresholds of |log2FC| ≥ 1 and *p* < 0.05. The intersecting genes of ANRGs and DEGs were then extracted (Figure 1A). These intersecting genes were analyzed using one-way Cox regression, resulting in the identification of 11 genes closely associated with the prognosis (Figure 1B). Eight overlapping genes were further filtered using six machine learning algorithms (Figure 1C–G). Finally, five genes were selected through multifactorial Cox regression analysis (Figure 1K).

### 3.2. Construction and Validation of the ANRS

**Figure 2:**
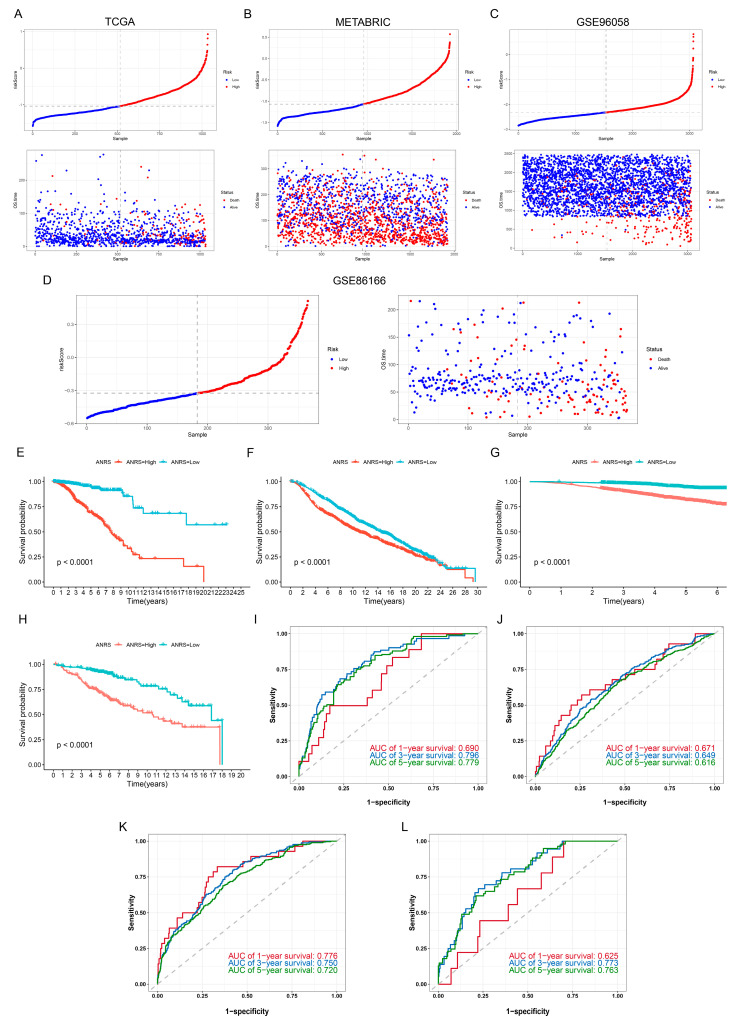
Construction of the ANRS. (**A**–**D**) Scatter plots of ANRS and survival status in TCGA-BRCA, METABRIC, GSE96058, and GSE86166. (**E**–**H**) Survival curves of high- and low-ANRS groups in TCGA-BRCA, METABRIC, GSE96058, and GSE86166. (**I**–**L**) ROC curves for predicting 1, 3, and 5-year survival for the ANRS in TCGA-BRCA, METABRIC, GSE96058, and GSE86166.

Among 14 machine learning algorithms, we selected the GBM algorithm with the highest C-index for constructing the ANRS in the TCGA-BRCA and METABRIC cohorts (Figure S1). Furthermore, we assessed the efficacy of the ANRS in the GSE96058 and GSE86166 cohorts using the same approach. BC patients were categorized into high-ANRS and low-ANRS groups based on the median of ANRS. An elevation in the ANRS was linked to a greater number of patient fatalities (Figure 2A–D). BC patients in the high-ANRS group exhibited a poorer prognosis across the TCGA-BRCA, METABRIC, GSE96058, and GSE86166 cohorts (Figure 2E–H). In TCGA-BRCA, the ANRS demonstrated AUC values of 0.690, 0.796, and 0.779 for predicting 1-, 3-, and 5-year overall survival (OS) in BC patients (Figure 2I). In the METABRIC cohort, the AUC values for predicting 1-, 3-, and 5-year OS were 0.671, 0.649, and 0.619 (Figure 2J). For the GSE96058 cohort, the ANRS yielded AUC values of 0.776, 0.750, and 0.720 for 1-, 3-, and 5-year OS prediction (Figure 2K). In the GSE86166 cohort, the AUC values for 1-, 3-, and 5-year OS predictions were 0.625, 0.773, and 0.763 (Figure 2L).

### 3.3. Relationship Between the ANRS and Clinical Features

**Figure 3:**
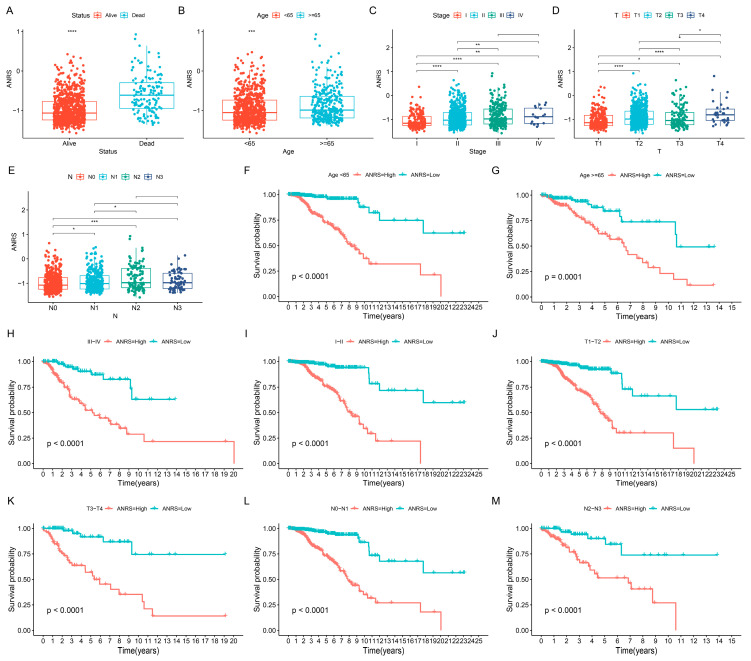
Relationship between ANRS and clinical characteristics. Box plots demonstrate the differences in ANRS across Status (**A**), Age (**B**), Stage (**C**), T stage (**D**), and N stage (**E**) in the TCGA-BRCA cohort. Survival curves for the TCGA-BRCA cohort for age <60 (**F**), age ≥60 (**G**), stage I–II (**H**), stage III–IV (**I**), T1–T2 (**J**), T3–T4 (**K**), N0–N1 (**L**), and N2–N3 (**M**). * *p* < 0.05; ** *p* < 0.01; *** *p* < 0.001; **** *p* < 0.0001.

To further evaluate the clinical significance of the ANRS, its association with clinical features was examined. The analysis indicated that BC patients who succumbed to the disease (Figure 3A) and those older than 65 years exhibited higher ANRS values (Figure 3B). Additionally, the ANRS varied significantly by stage, T, and N (Figure 3C–E). Clinical features in the TCGA-BRCA cohort were categorized into subgroups to assess the predictive capability of the ANRS across various patient groups. The results demonstrated that ANRS could effectively predict the prognosis of patients stratified by age (<65 years and ≥65 years), stage (I–II and III–IV), T (T1–T2 and T3–T4), and N (N0–N1 and N2–N3) (Figure 3F–M).

### 3.4. Comparison Between ANRS and the Published Prognostic Signatures

We compared the ANRS with previously published prognostic signatures, and information on these signatures is presented in Table S3. We constructed these signatures in the TCGA-BRCA, METABRIC, GSE96058, and GSE86166 cohorts following the methodology used in previous studies. We evaluated the C-index as well as the AUC values for these signatures and compared them with the ANRS. The ANRS had the highest AUC values and C-index in all cohorts (Figure S2).

### 3.5. Construction of the Nomogram Associated with Aoikis

**Figure 4:**
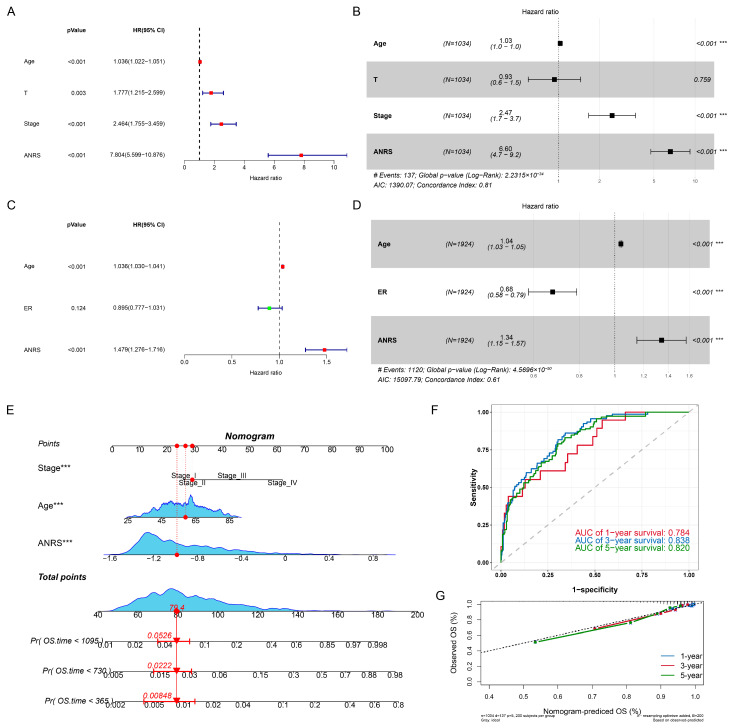
Nomogram construction. (**A**–**D**) Univariate and multivariate Cox regression analyses were performed to evaluate the independence of the ANRS. (**E**) A nomogram was developed based on the ANRS and clinical characteristics. (**F**) ROC curves were plotted to assess the performance of the nomogram. (**G**) Calibration curves were generated to compare the predicted survival probabilities with the actual outcomes. *** *p* < 0.001.

The results of unifactorial Cox and multifactorial Cox analyses in the TCGA-BRCA and METABRIC cohorts demonstrated that the ANRSs were independent predictors of other clinical features (Figure 4A–D). In TCGA-BRCA, we developed a nomogram that integrated age and stage to predict the prognosis of BC patients (Figure 4E). To assess the accuracy of the nomogram’s predictions, we generated ROC curves. The AUC values for 1-, 3-, and 5-year predictions were 0.784, 0.836, and 0.820 (Figure 4F). Additionally, the calibration curves demonstrated that the predicted survival probabilities from the nomogram closely matched the observed survival rates (Figure 4G).

### 3.6. Functional Analysis of the Prognostic Signature

The GO analysis revealed that differentially expressed genes were primarily enriched in areas such as cell adhesion, cell killing, and immune responses (Figure S3A). According to KEGG analysis, the enriched pathways include the B cell receptor signaling pathway, chemokine signaling pathway, and cytokine–cytokine receptor interaction (Figure S3B). Consistent with these findings, GSVA-GO results indicated significant differences between high-ANRS and low-ANRS groups in biological processes such as T cell activation involved in immune response, regulation of cell killing, and programmed cell death involved in cell development (Figure S3C). As depicted in Figure S3D, there are distinctions between the two ANRS groups in pathways such as the B cell receptor signaling pathway, focal adhesion, and ECM receptor interaction. These findings suggest that prognostic features are closely related to a variety of biological functions in breast cancer.

### 3.7. Relationship between ANRS and TME

**Figure 5:**
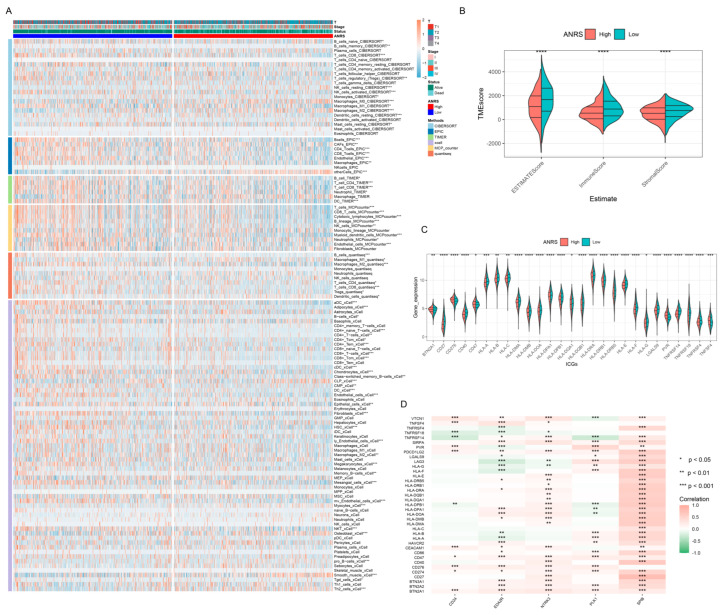
Differences in immune cell infiltration between high- and low-ANRS groups. (**A**) A heatmap illustrating the variation in immune cell infiltration levels calculated using five algorithms between the different ANRS groups. (**B**) Comparison of tumor microenvironmental scores (Immune, Stromal, and ESTIMATE scores) between the high- and low-ANRS groups. (**C**) Differential expression of immune checkpoints between the high- and low-ANRS groups. (**D**) Correlation analysis between the prognostic ANRGs and immune checkpoints. * *p* < 0.05; ** *p* < 0.01; *** *p* < 0.001; **** *p* < 0.0001.

Figure 5A illustrates the variation in immune cell infiltration between the high-ANRS and low-ANRS groups. As depicted in Figure 5B, significant differences were observed in ESTIMATEScore, ImmuneScore, and StromalScore between these groups. Immune checkpoint genes (ICGs), which play a critical role in the TME, were also analyzed in relation to the ANRS. The results indicated that the majority of ICGs were significantly different between the high-ANRS and low-ANRS groups (Figure 5C). Additionally, genes associated with the ANRS were found to be notably correlated with immune checkpoints (Figure 5D).

### 3.8. Immunotherapy Prediction and Drug Sensitivity Analysis

**Figure 6:**
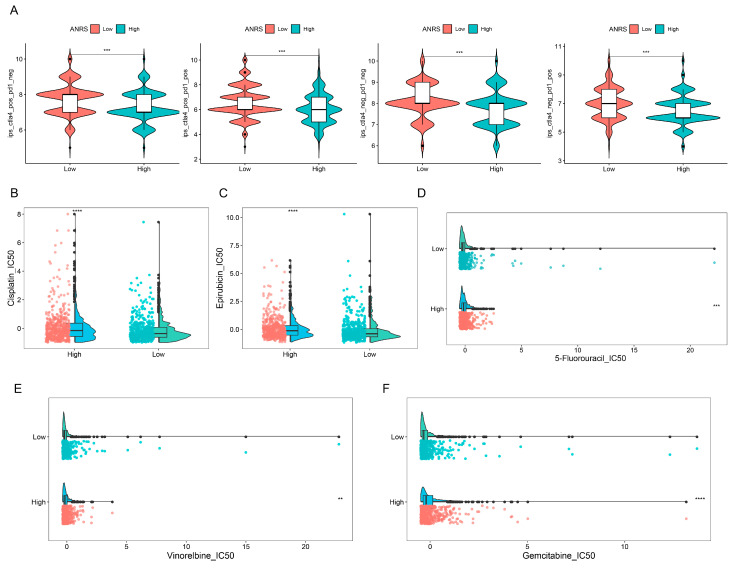
Immunotherapy and drug sensitivity. (**A**) Differences in immunophenotype score (IPS) between different ANRS groups. IPS predicts patient response to anti-PD-1/PD-L1 or anti-CTLA4 therapy. Analysis of sensitivity differences (IC50) of chemotherapeutic drugs between high- and low-ANRS groups. (**B**–**F**) cisplatin, epirubicin, 5-Fluorouracil, vinorelbine, gemcitabine. ** *p* < 0.01; *** *p* < 0.001; **** *p* < 0.0001.

Since the ANRSs were closely related to the TME, the response to immunotherapy was further analyzed in the high-ANRS and low-ANRS groups. Compared to the high-ANRS group, the IPS scores were higher in the low-ANRS group (Figure 6A). The drug sensitivity analysis results showed that tumor cells in the low-ANRS group were more sensitive to Cisplatin (Figure 6B), Epirubicin (Figure 6C), 5-Fluorouracil (Figure 6D), Vinorelbine (Figure 6E), and Gemcitabine (Figure 6F). Furthermore, the low-ANRS group was more sensitive to Ribociclib (CD4/6 inhibitor), Palbociclib (CD4/6 inhibitor), Sorafenib (multi-kinase inhibitor), Erlotinib (EGFR inhibitor), Olaparib (PARP inhibitor), and Niraparib (PARP inhibitor) (Figure S4A–F).

### 3.9. Single-Cell Analysis and Spatial Transcriptomics

**Figure 7:**
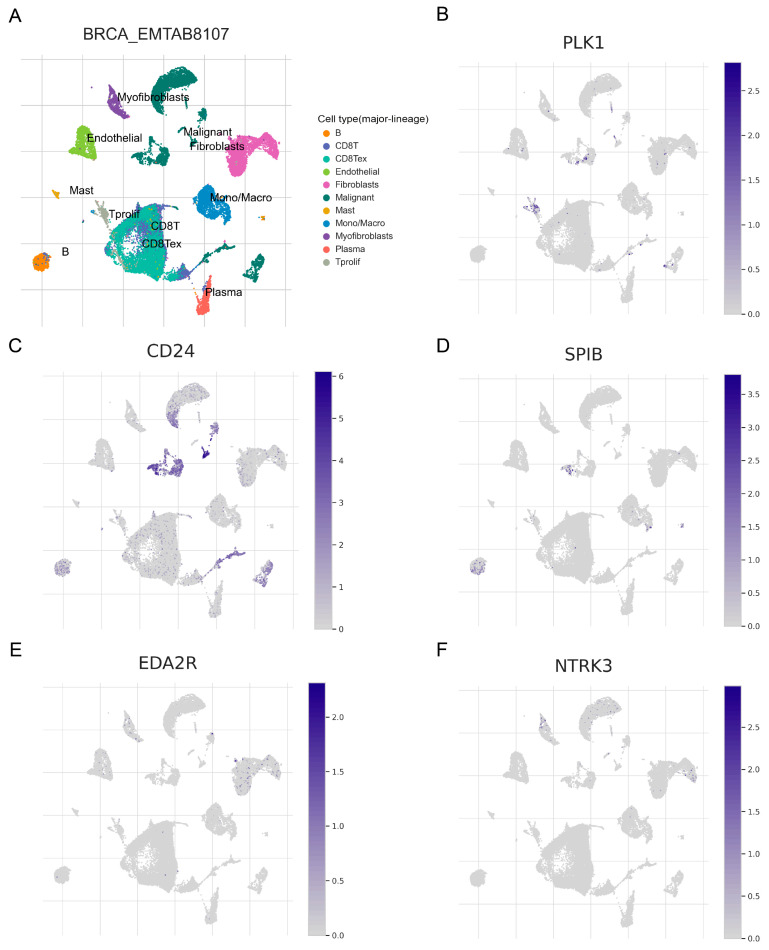
Single-cell analysis of prognostic ANRGs. (**A**) Different cell types within the EMTAB8107 dataset. Expression levels of PLK1 (**B**), SPIB (**C**), CD24 (**D**), EDA2R (**E**), and NTRK3 (**F**) in different cells.

**Figure 8:**
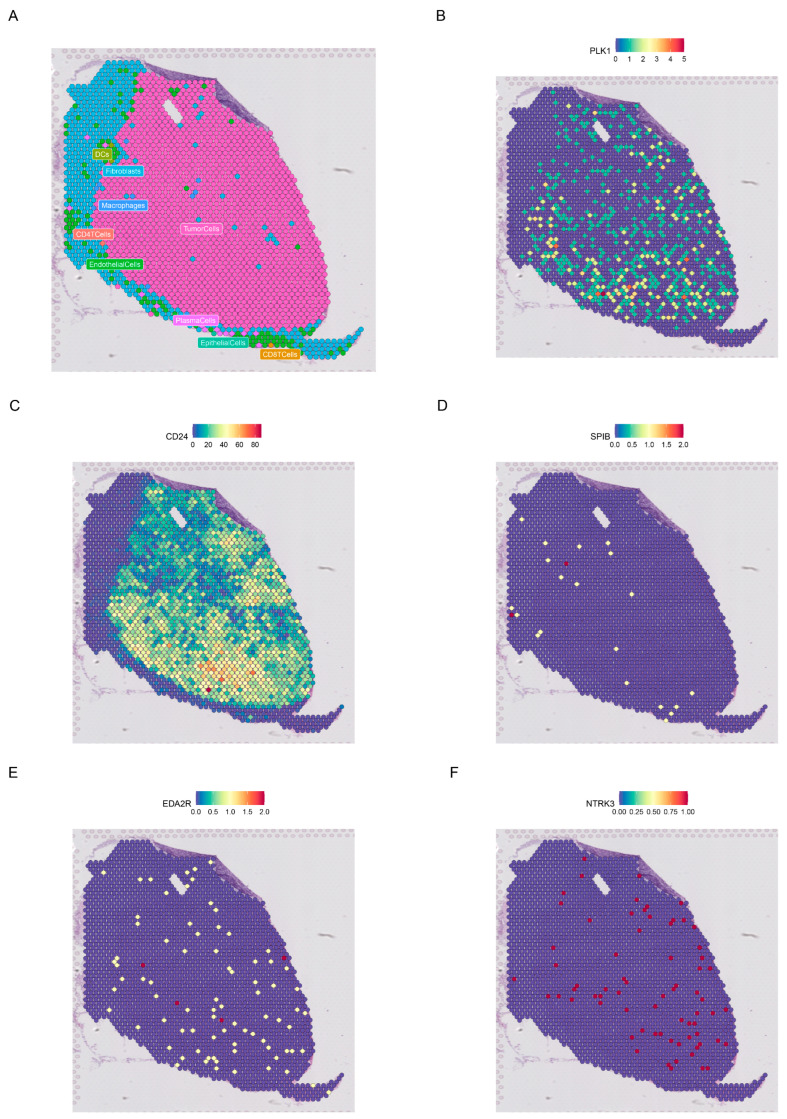
Spatial transcriptomic analysis of prognostic ANRGs. (**A**) Distribution of different cells in GSE203612-GSM6177603-NYU-BRCA2. (**B**–**F**) Distribution of PLK1, CD24, SPIB, EDA2R, and NTRK3 in different cells.

Figure 7A displays the diverse cell types within the EMTAB8107 dataset. Single-cell analysis results indicate that SPIB is predominantly enriched in malignant cells and B cells (Figure 7B). CD24 is abundantly expressed in malignant cells (Figure 7C). PLK1 shows high expression in malignant cells and Tprolif cells (Figure 7D). Additionally, EDA2R (Figure 7E) and NTRK3 (Figure 7F) exhibit lower expression across various cells. Figure 8A illustrates the spatial distribution of different cells in GSE203612-GSM6177603-NYU_BRCA2. PLK1 and CD24 are highly expressed in malignant cells, while SPIB, EDA2R, and NTRK3 exhibit lower expression across different cell types (Figure 8B–F).

### 3.10. Prediction of Potential Drugs

**Figure 9:**
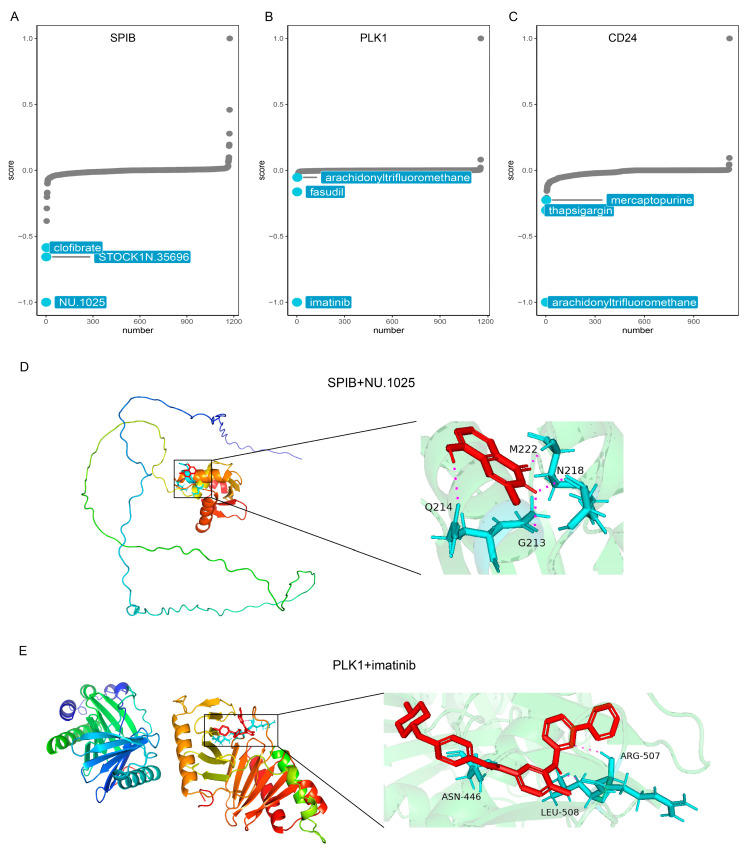
Prediction of potential drugs. (**A**–**C**) CMap analysis reveals the top three potential drugs targeting SPIB, PLK1, and CD24. (**D**,**E**) Diagrams of the molecular docking models of drugs with proteins, their active sites, and binding distances.

Utilizing the eXtreme Sum (XSum), we identified targeted drugs for the three highly expressed genes (SPIB, PLK1, and CD24) based on CMap scores. Figure 9A–C display the top three targeted drugs for SPIB, PLK1, and CD24, respectively. Figure 9D presents the molecular docking model of the SPIB protein with NU.1025, highlighting the active site and binding distance. Figure 9E illustrates the molecular docking model of the PLK1 protein with imatinib, detailing the active site and binding distance. 

### 3.11. Validation of the Expression Level of the Prognostic ANRGs

**Figure 10:**
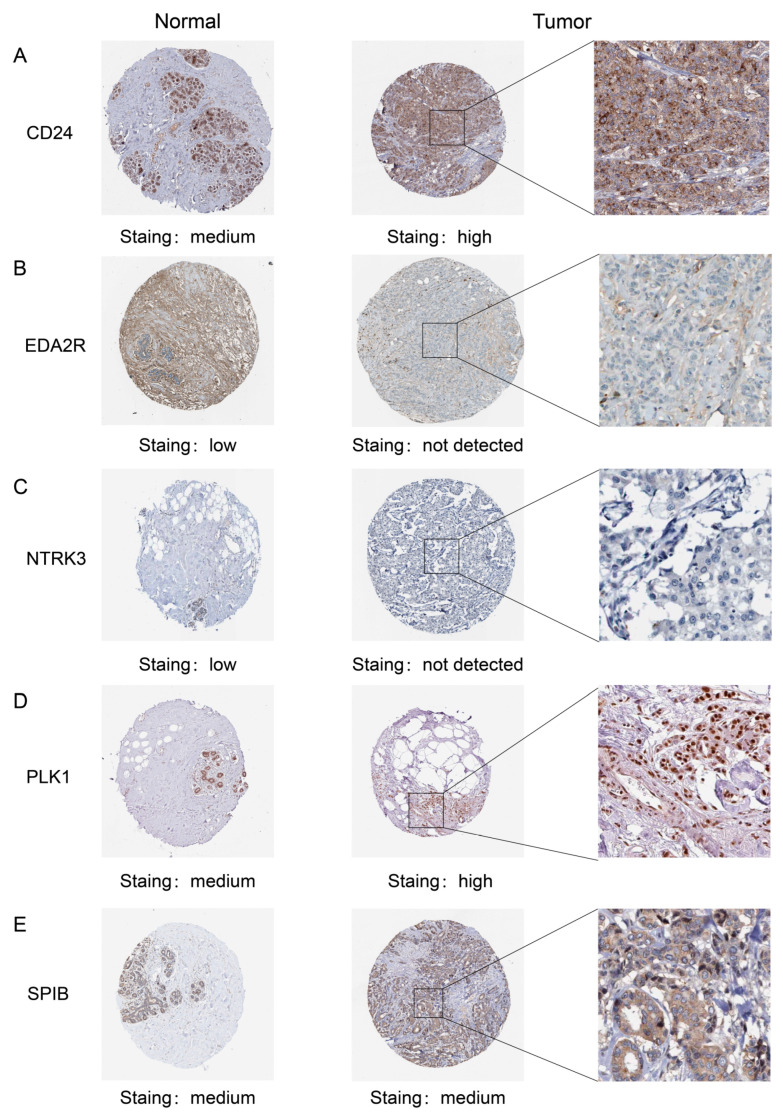
IHC images of the prognostic ANRGs. Protein staining levels of CD24 (**A**), EDA2R (**B**), NTRK3 (**C**), PLK1 (**D**), and SPIB (**E**) in normal and BC tissues.

**Figure 11:**
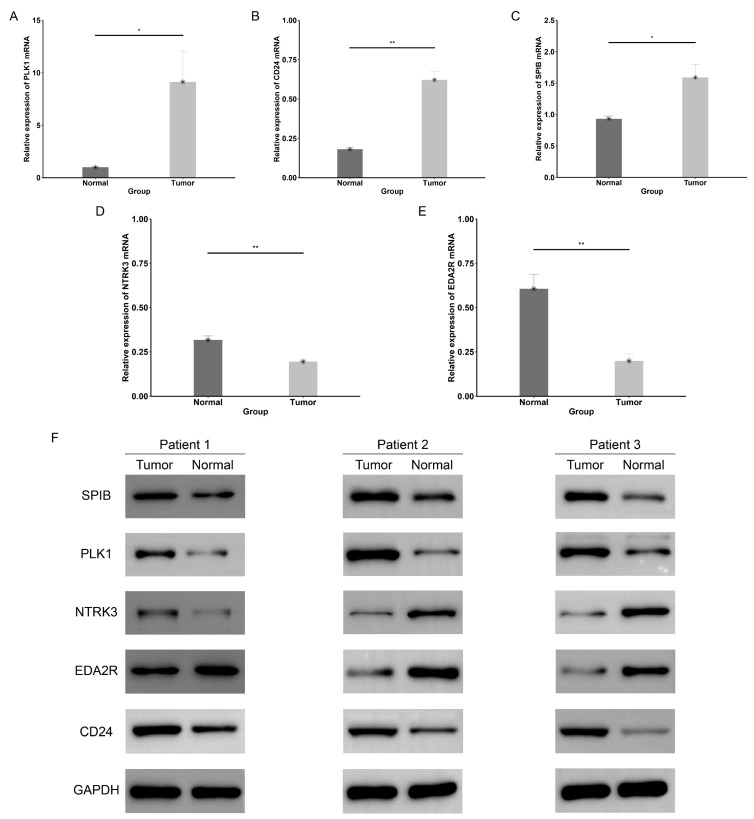
Experimental validation of the prognostic ANRGs. (**A**–**E**) The mRNA expression levels of the prognostic ANRGs in breast cancer tissues and paracancerous tissues. (**F**) Protein expression levels of the prognostic ANRGs in breast cancer tissues and paracancerous tissues. * *p* < 0.05; ** *p* < 0.01.

The prognostic ANRG expression levels were verified across multiple datasets, including tissues and cell lines, as illustrated in Figure S5A–L. Additionally, we observed higher expression levels of *PLK1* (Figure S5M) and *CD24* (Figure S5N) in the blood of BC patients. Immunohistochemistry (IHC) images from the HPA database were downloaded to compare the expression of prognostic ANRGs in normal and tumor tissues. As demonstrated in Figure 10, the proteins PLK1, SPIB, and CD24 show darker staining in tumor tissues, while NTRK3 and EDA2R exhibit lighter staining in tumor tissues. The mRNA expression of *SPIB*, *PLK1*, and *CD24* is upregulated in clinical tumor tissues, whereas *NTRK3* and *EDA2R* mRNA expression is downregulated (Figure 11A–E). In clinical tumor tissues, protein expression of SPIB, PLK1, and CD24 was up-regulated, while protein expression of NTRK3 and EDA2R was down-regulated (Figure 11F).

### 3.12. Identification of PLK1 as a Diagnostic Marker

**Figure 12:**
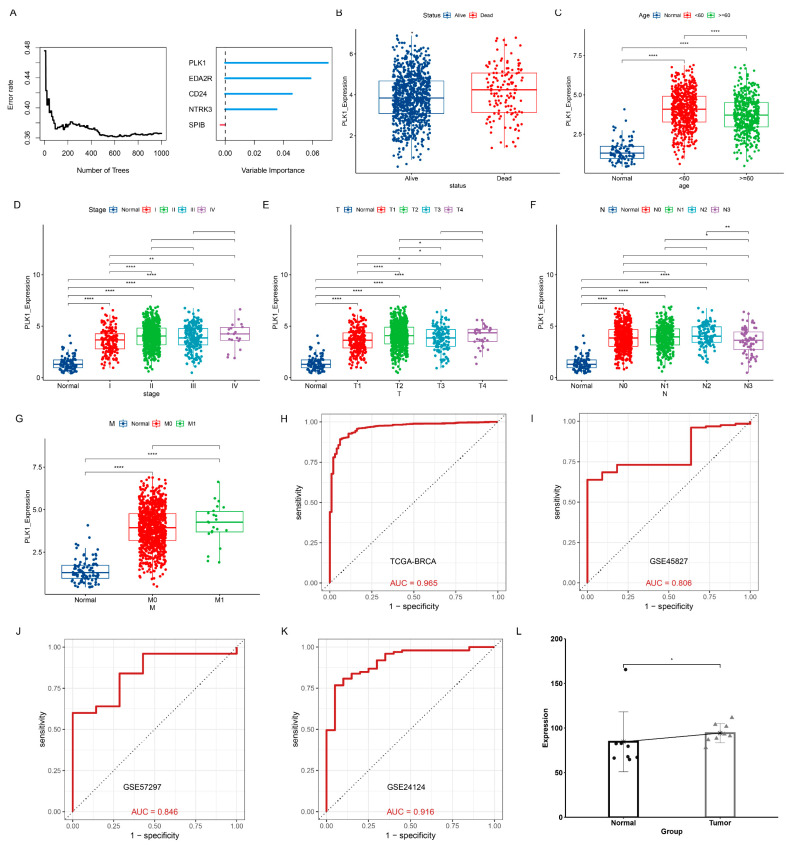
Comprehensive analysis of PLK1. (**A**) Error rate plots and variable significance plots of the random forest algorithm. Differential expression of PLK1 in different status (**B**), age (**C**), stage (**D**), T-stage (**E**), N-stage (**F**), and M-stage (**G**). (**H**–**K**) Diagnostic ROC for PLK1 in the TCGA-BRCA, GSE45827, GSE57297, and GSE24124 cohorts. (**L**) Expression levels of PLK1 in the serum of normal subjects and breast cancer patients. * *p* < 0.05, ** *p* < 0.01, **** *p* < 0.0001.

The results from the random survival forest algorithm indicate that PLK1 is the core gene among the prognostic ANRGs (Figure 12A). Furthermore, significant differences in *PLK1* expression levels were observed across different statuses, ages, stages, T stages, N stages, and M stages (Figure 12B–G). Therefore, we further analyzed the diagnostic value of PLK1. The results of ROC curves showed that PLK1 was effective in distinguishing between normal and BC patients in the TCGA-BRCA, GSE45827, GSE57297 and GSE24124 datasets (Figure 12H–K). Moreover, PLK1 also demonstrates high diagnostic accuracy for different BC subtypes and pathological stages in the TCGA-BRCA dataset (Figure S6). ELISA results further demonstrate that PLK1 expression is elevated in the blood of breast cancer patients compared to healthy controls (Figure 12L). Meta-analysis findings suggest that PLK1 serves as a risk factor for breast cancer (*p* = 0.28, I^2^ = 22%, Figure S7A). Importantly, PLK1 is strongly linked to processes such as the cell cycle, cell proliferation, DNA damage, and DNA repair (Figure S7B).

## 4. Discussion

Breast cancer is a multifaceted disease defined by distinct somatic mutations and alterations in gene and protein expression [38]. Due to the complexity of BC, the identification of accurate prognostic biomarkers is essential for better monitoring of cancer progression, metastasis, and recurrence [39]. Disruption of cell death mechanisms is a key factor in the progression of breast cancer. Generally, cells maintain the homeostasis of tissues through cell death mechanisms such as apoptosis, which avoids the proliferation of abnormal cells [40]. However, in breast cancer, cancer cells often evade immune surveillance by inhibiting apoptosis-related genes or activating anti-apoptotic signaling pathways, resulting in uncontrolled proliferation [41].

Anoikis resistance, the ability of cells to avoid apoptosis when detached from the extracellular matrix, is believed to promote carcinogenesis and progression, while inducing anoikis in cancer cells inhibits their tumorigenicity and metastatic potential [42,43,44]. A considerable number of studies have established the critical role of anoikis-related genes in breast cancer. For instance, ZNF367 has been shown to suppress the Hippo pathway and activate Yes-associated protein 1 (YAP1), contributing to anoikis resistance in BC [45]. Additionally, another study demonstrated that ErbB2 inhibits the upregulation of BLNK by reducing the levels of the tumor cell transcription factor IRF6, thus facilitating breast tumor growth [46]. Thus, ANRGs are potential therapeutic targets in breast cancer.

With the advancement of medical technology, especially the rapid development of machine learning and artificial intelligence techniques, new tools have been developed for the understanding, diagnosis, and treatment of cancer. Machine learning, as a technique capable of learning patterns and regularities from data, has shown great potential in cancer research [47]. It can process and analyze large amounts of biomedical data, including genomic data, medical images, and patients’ clinical records, to reveal cancer biomarkers, predict treatment outcomes, and develop personalized treatment plans [48,49,50,51]. In this study, we developed the ANRS using various machine learning algorithms and validated its prognostic significance across additional GEO cohorts. The ANRS shows robust predictive performance in multiple cohorts and can well predict the prognosis of patients with different clinicopathologic features of BC. Then, we generated a new nomogram by combining clinicopathologic features and ANRS of BC patients. Calibration and ROC curves demonstrated that the nomogram exhibited strong predictive accuracy.

With the deeper understanding of cell death mechanisms in breast cancer, researchers have progressively developed prognostic models based on cell death-related genes. For example, a recent study developed a ferroptosis-related prognostic model with good predictive ability, which provides new ideas for the treatment of gastric cancer patients in the clinic [52]. In this study, we collected ten signatures in breast cancer, three of which are related to anoikis and the others to the tumor microenvironment. The ANRS had a higher C-index and AUC values than other characteristics in different cohorts, suggesting that ANRS is an accurate and robust prognostic indicator in breast cancer.

The ANRS consists of five ANRGs: *SPIB*, *CD24*, *NTRK3*, *EDA2R*, and *PLK1*, all of which have been extensively linked to cancer. One study demonstrated that SPIB facilitates anoikis resistance by enhancing autolysosomal processes in lung cancer cells [53]. Furthermore, researchers have shown that SPIB can prolong the lifespan of memory B cells by modulating the expression of B cell-related genes [54]. Our findings also revealed that SPIB was highly enriched in B cells. CD24 was found to be highly expressed in tumor tissues, contributing to immune evasion. Inhibition of CD24 resulted in reduced macrophage-dependent tumor growth and extended survival time in vivo [55]. Specific CAR-T cells targeting CD24 have been shown to exhibit significant anti-tumor activity and hold potential for use in the treatment of triple-negative breast cancer [56]. In the current study, we also observed that CD24 was highly expressed in malignant cells. Hsa-circ-0009172 suppressed the expression of *NTRK3* by acting as a sponge for miR-485-3p, which in turn inhibited the proliferation, invasion, and migration capabilities of gastric cancer cells, as well as the expression of epithelial-mesenchymal transition-related proteins [57]. EDA2R (XEDAR) is a tumor suppressor that prevents apoptosis and anoikis by regulating tumor progression [58]. Polo-like kinase 1 (PLK1) is a crucial mitotic kinase that is frequently overexpressed in numerous cancers, where it facilitates carcinogenesis and accelerates disease progression [59,60]. Consistent with previous studies [61], our findings also indicate that BC patients with high *PLK1* expression have a worse prognosis. Additionally, PLK1 is closely associated with immunotherapy, chemotherapy, and radiotherapy for cancer [62]. Inhibition of PLK1 decreased radiation-induced ROS and autophagy levels, thereby enhancing the sensitivity of breast cancer cells to radiation [63]. Another study demonstrated that inhibiting PLK1 led to the upregulation of PD-L1 expression in pancreatic ductal adenocarcinoma, which enhanced anti-tumor immunity and increased tumor sensitivity to immunotherapy [64]. Finally, the results of RT-PCR and WB aligned with the differential expression levels observed in the database, further validating the predictive accuracy of the prognostic signature.

Given the above roles of ANRGs in cancer, we predicted potential target drugs for three highly expressed genes using the CMap method. The results of molecular docking showed that NU.1025 and imatinib could serve as potential inhibitors of SPIB and PLK1, respectively. Previous studies have identified the inhibitory effects of these small-molecule compounds in cancer [65,66,67,68]. Our results elucidate the potential molecular mechanisms of cancer inhibition by these drugs, but further experiments are needed for validation.

To further explore the underlying biological mechanisms of the prognostic signature, we performed functional enrichment analysis. The high-ANRS group was found to be strongly associated with biological processes that promote tumor development, including the positive regulation of protein tyrosine kinase activity, the production of transforming growth factor β (TGF-β), and the involvement in focal adhesion. Various studies have indicated that the abnormal activation of protein tyrosine kinases, triggered by diverse factors, can redirect cellular functions towards abnormal growth states, culminating in tumorigenesis [69]. TGF-β is recognized as a pivotal factor in promoting epithelial-mesenchymal transition (EMT), enabling immune evasion, and facilitating metastasis in cancer’s advancement [70,71]. Furthermore, the dysregulation in cell adhesion signaling is often implicated in numerous cellular dysfunctions, such as enhanced cell survival, migration, and the invasion of cancer cells [72]. In contrast, the low-ANRS group exhibited a primary enrichment in processes related to cellular apoptosis and immune responses, including T cell activation, programmed cell death essential for cell development, and lymphocyte activation, all of which are known to counteract cancer development [73,74,75].

Next, we analyzed the relationship between the ANRS and the tumor microenvironment, which encompasses tumor cells, immune cells, and cytokines. These components are categorized as either anti-tumor or pro-tumor, and their interactions ultimately influence the direction of tumor immunity [76]. Our analysis showed that the low-ANRS group had a high level of immune cell infiltration and an elevated immuneScore. These results suggest that the tumor microenvironment in the low-ANRS group is linked to the inhibition of cancer progression. In recent years, the introduction of immunotherapy has transformed cancer treatment and reinvigorated the domain of tumor immunology [77]. A range of immunotherapeutic approaches, such as immune checkpoint inhibition, vaccination, and adoptive cell transfer, have been thoroughly investigated in the clinical treatment of breast cancer, especially in individuals with triple-negative breast cancer [78]. Higher TIDE and IPS scores were observed in the low-ANRS group, indicating that these patients might exhibit a more favorable response to immunotherapy.

Currently, the main clinically recommended adjuvant therapies for breast cancer include chemotherapy (anthracycline, taxane, platinum, and capecitabine), targeted therapies (Her2 inhibitor, CD4/6 inhibitor), endocrine therapies (tamoxifen, aromatase inhibitor, ovarian ablation/suppression, and bisphosphonates), and radiotherapy [79,80]. Radiotherapy is commonly administered following breast-conserving surgery (including whole breast, partial breast, and localized lymph node treatment) or mastectomy [81]. In this study, we found that the low-ANRS group was more susceptible to chemotherapy-related drugs (cisplatin, epirubicin, 5-Fluorouracil), CD4/6 inhibitors (ribociclib, palbociclib), EGFR inhibitors (erlotinib), and PARP inhibitors (olaparib, niraparib). A recent study demonstrated that the combination of ribociclib and a nonsteroidal aromatase inhibitor notably enhanced invasive disease-free survival in patients with stage II or III early breast cancer, both HER2-positive and HER2-negative [82]. These results further support the potential value of the ANRS as a biomarker related to clinical response to treatment in breast cancer.

Through the random forest algorithm, we identified *PLK1* as a potential core gene among the five key genes. We further explored the role of PLK1 in breast cancer and found that it could effectively distinguish normal individuals from breast cancer patients across different subtypes and pathological stages. Additionally, we observed that *PLK1* was overexpressed in breast cancer tissues, peripheral blood, and cell lines, highlighting its diagnostic value. We also verified the elevated expression levels of PLK1 in the blood of breast cancer patients compared to normal subjects. These findings suggest that PLK1 can serve as a potential blood diagnostic marker for breast cancer patients.

While the ANRS has yielded satisfactory results in forecasting the outcomes for BC patients, several limitations of the model must be acknowledged. Firstly, the machine learning approach may be influenced by biases related to the selection and quality of input data, such as potential overfitting due to small sample sizes or unbalanced datasets. Despite employing cross-validation and external validation cohorts, the ANRS still needs to be tested for its clinical utility in real cohorts. Lastly, further experimental research is necessary to validate the relationships among the genes identified and to elucidate the biological mechanisms underlying these associations.

## 5. Conclusions

In summary, we have developed an anoikis-related prognostic signature for breast cancer that effectively predicts patient outcomes. It can also assess the tumor microenvironment of BC patients as well as their response to immunotherapy and chemotherapy. Additionally, we have identified PLK1 as a promising blood-based diagnostic marker for BC. Our study offers new insights for the personalized treatment of BC patients.

## Data Availability

The dataset provided in this study can be downloaded from the online website. TCGA-BRCA: https://portal.gdc.cancer.gov/ (accessed on 25 February 2024). METABRIC: https://www.cbioportal.org/ (accessed on 27 February 2024). GEO: https://www.ncbi.nlm.nih.gov/geo/ (accessed on 28 March 2024).

## Supplementary Materials

The following supporting information can be downloaded at: https://www.mdpi.com/article/10.3390/genes15111458/s1, Figure S1: GBM was selected as the modeling method among 14 ML methods for scoring; Figure S2: Comparison between ANRS and the published prognostic signatures; Figure S3: Functional analysis of the ANRS; Figure S4: Sensitivity analysis (IC50) of targeted drugs between high- and low-ANRS groups; Figure S5: Validation of the prognostic ANRGs across different datasets; Figure S6: Diagnostic value of PLK1; Figure S7: Comprehensive analysis of PLK1; Table S1: Basic information on GEO datasets; Table S2: Basic information on clinical data; Table S3: The list of anoikis-related genes; Table S4: Primer sequences; Table S5: Other published prognostic signatures.
