# Supplementary material for: Integration of Machine Learning and Experimental Validation to Identify Anoikis-Related Prognostic Signature for Predicting the Breast Cancer Tumor Microenvironment and Treatment Response
Source: Genes (Basel). 2024 Nov 12;15(11):1458. doi: 10.3390/genes15111458 (PMC11594124; doi:10.3390/genes15111458)
Supplement: Supplementary file 1 [file genes-15-01458-s001.zip › Supplementary Figures.pdf]

Supplementary Figures

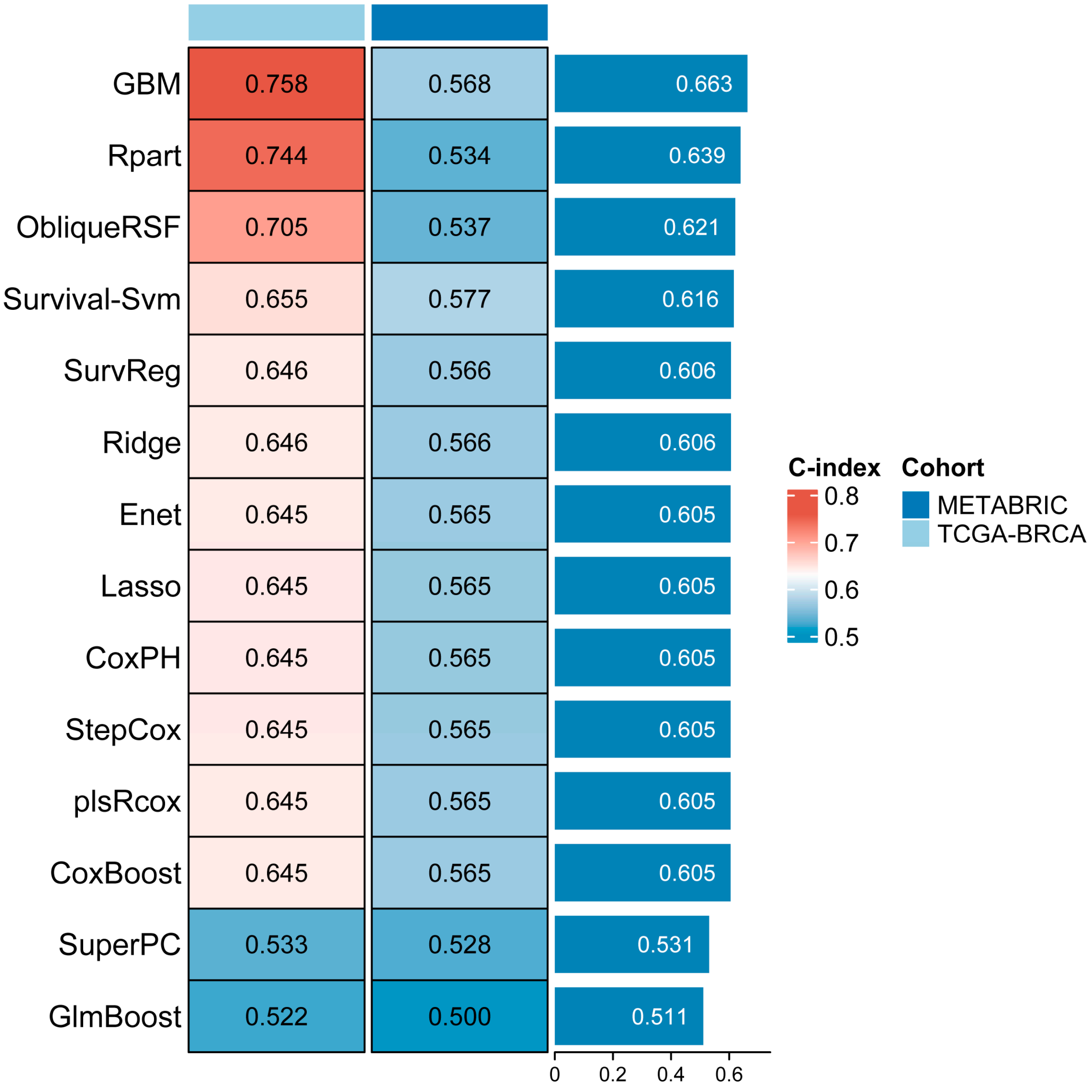

Figure S1 GBM was selected as the modeling method among 14 ML methods for scoring.

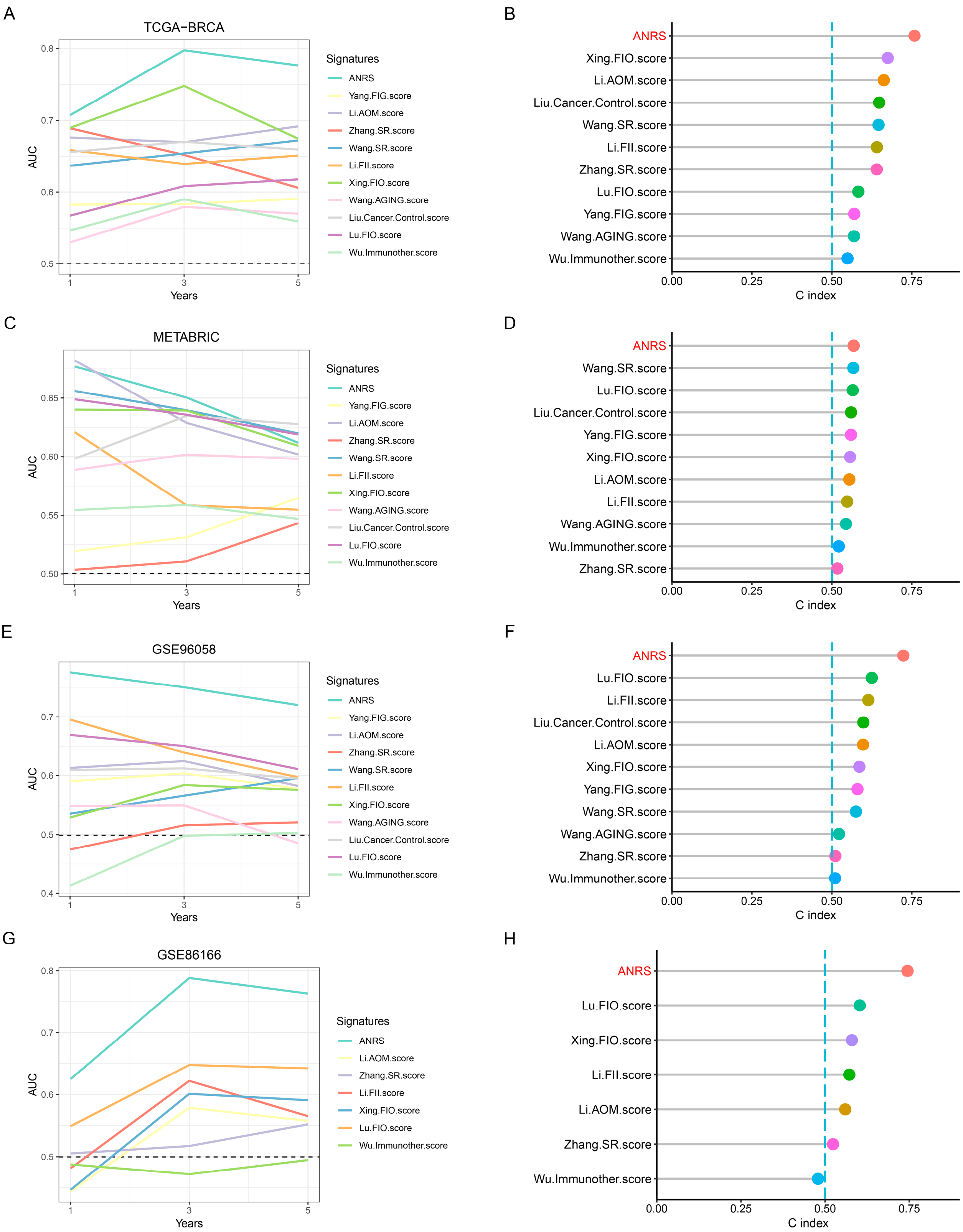

Figure S2 Comparison between ANRS and the published prognostic signatures. (**A, C, E**) AUC values comparison of ANRS and 10 previously published signatures in the TCGA-BRCA, METABRIC, and GSE96058 cohorts. (**G**) AUC values comparison of risk score and 6 previously published signatures in the GSE86166 cohort. (**B, D, F**) C-index comparison of ANRS and 10 previously published signatures in the TCGA-BRCA, METABRIC, and GSE96058 cohorts. (**H**) C-index comparison of ANRS and 6 previously published signatures in the GSE86166 cohort.

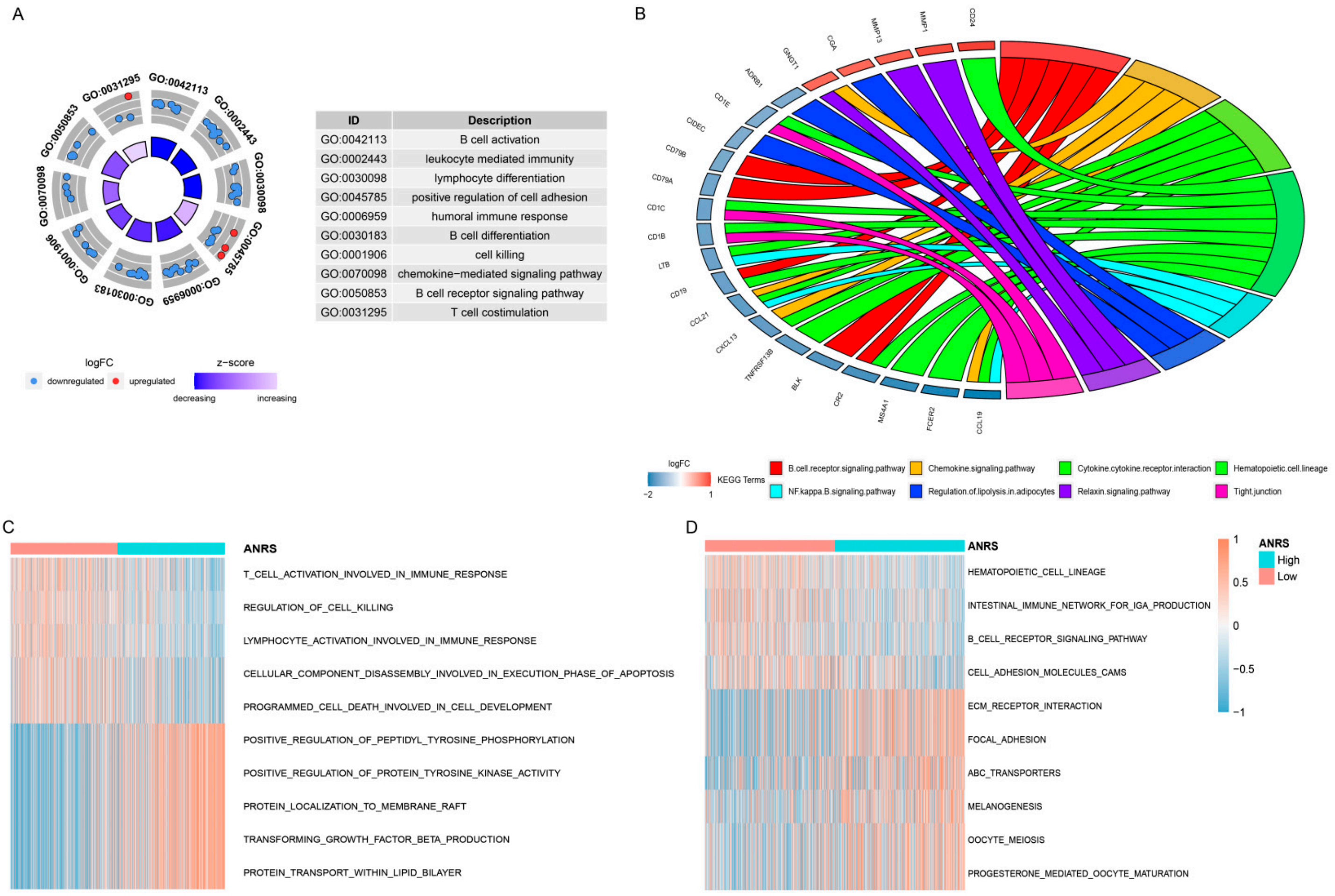

Figure S3 Functional analysis of the ANRS. (**A**, **B**) GO analysis and KEGG analysis of differentially expressed genes between high and low ANRS groups. Biological processes (**C**) and pathways (**D**) differing between high and low ANRS groups.

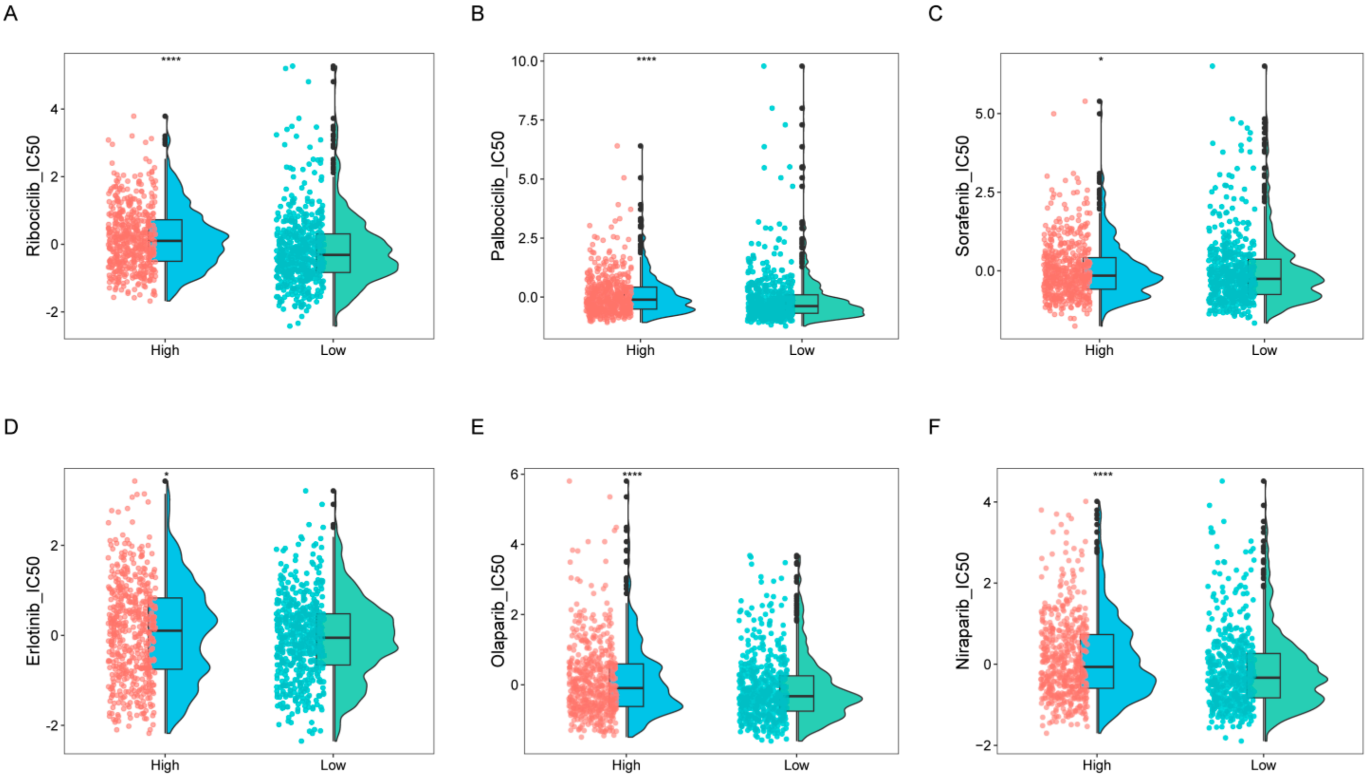

Figure S4 Sensitivity analysis (IC50) of targeted drugs between high- and low-ANRS groups. (**A-F**) Ribociclib, Palbociclib, Sorafenib, Erlotinib, Olaparib, Niraparib. * $P < 0.05$; ** $P < 0.01$; *** $P < 0.001$; **** $P < 0.0001$.

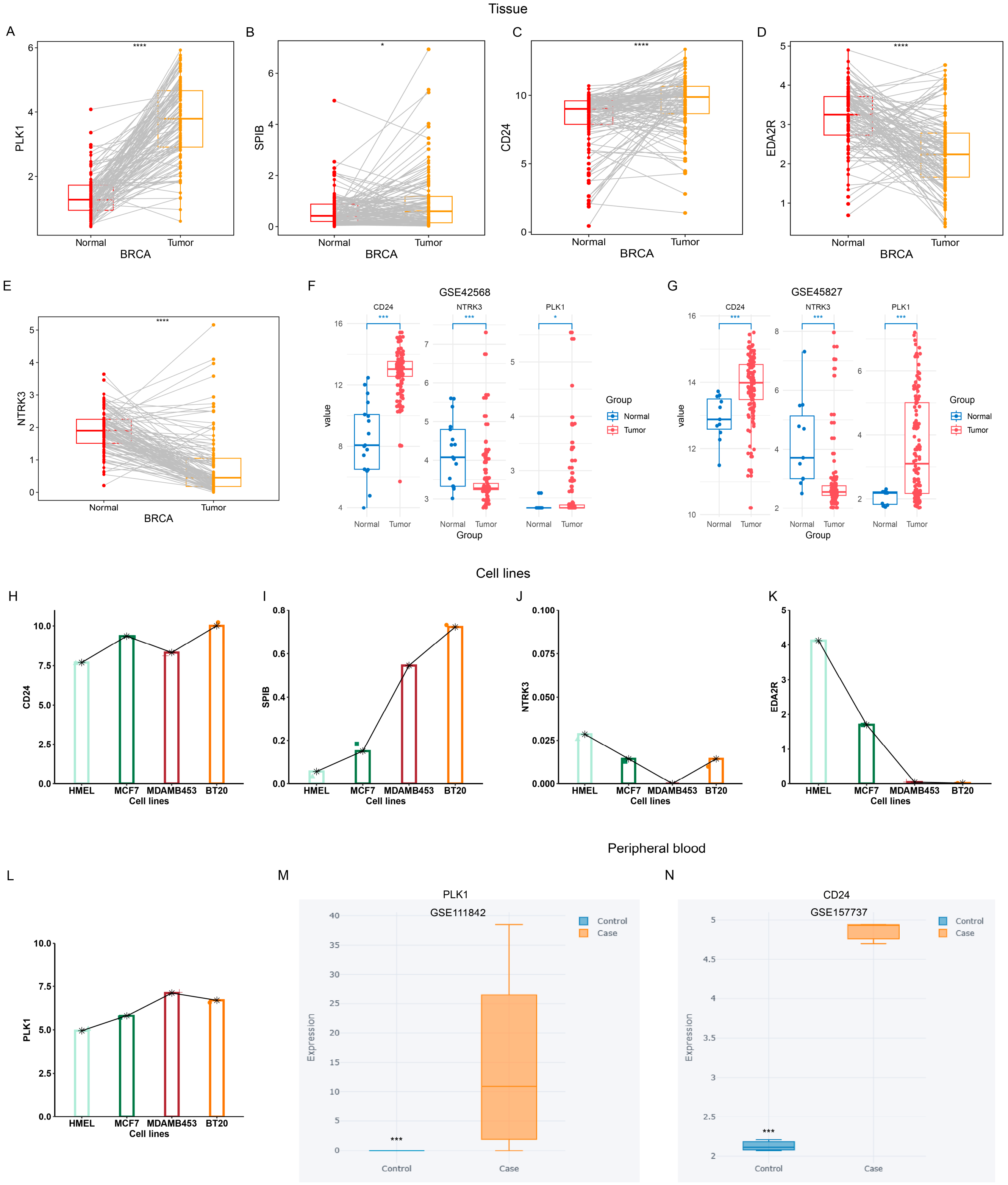

Figure S5 Validation of the prognostic ANRGs across different datasets. (**A-E**) Expression levels of PLK1, SPIB, CD24, EDA2R, and NTRK3 in BC tissues and adjacent normal tissues in the TCGA-BRCA cohort. (**F-G**) Expression levels of CD24, NTRK3, and PLK1 in GSE42568 and GSE45827. (**H-L**) Expression of PLK1, SPIB, CD24, EDA2R, and NTRK3 in cell lines (HMEL, MCF7, MDAMB453, and BT20) in the CCLE database. (**M**) Expression level of PLK1 in the blood of BC patients in the GSE111642 dataset. (**N**) Expression level of CD24 in the blood of BC patients in the GSE157737 dataset. * P < 0.05; ** P < 0.01; *** P < 0.001; **** P < 0.0001.

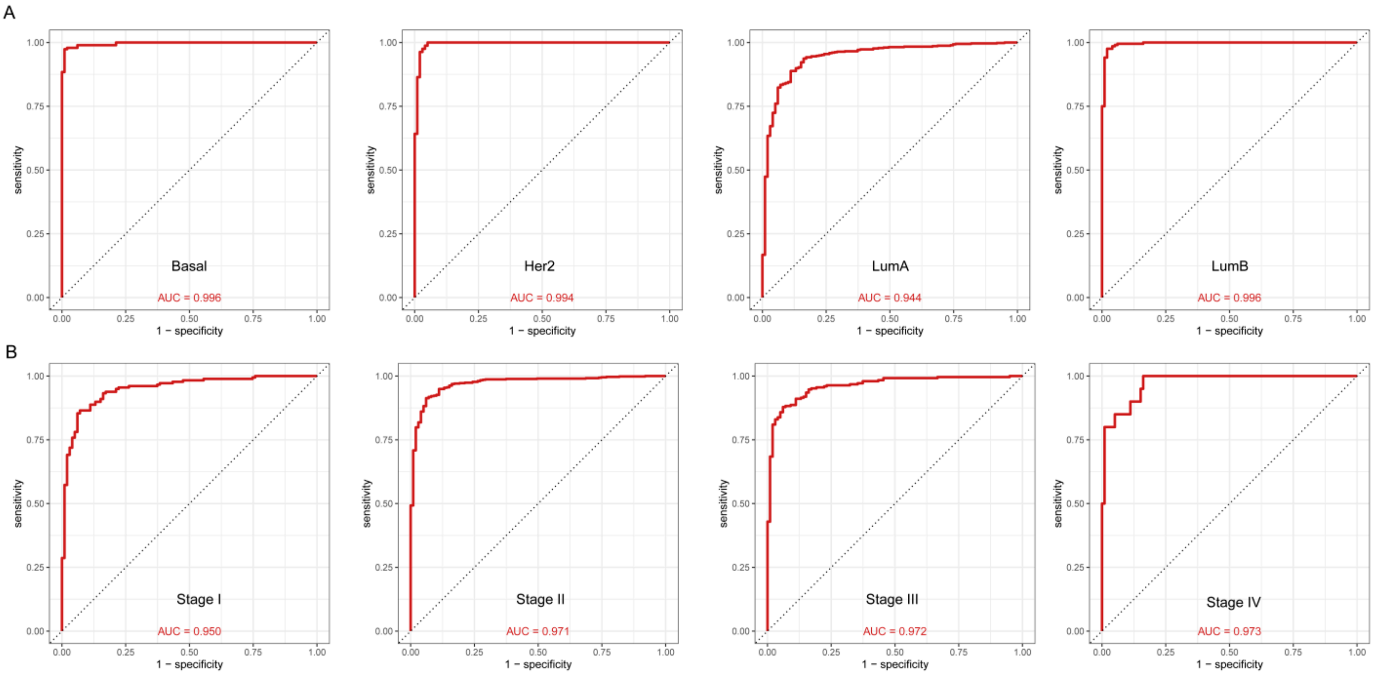

Figure S6 Diagnostic value of PLK1. Diagnostic ROC curves of PLK1 across different molecular subtypes (**A**) and different pathological stages (**B**) in the TCGA-BRCA cohort.

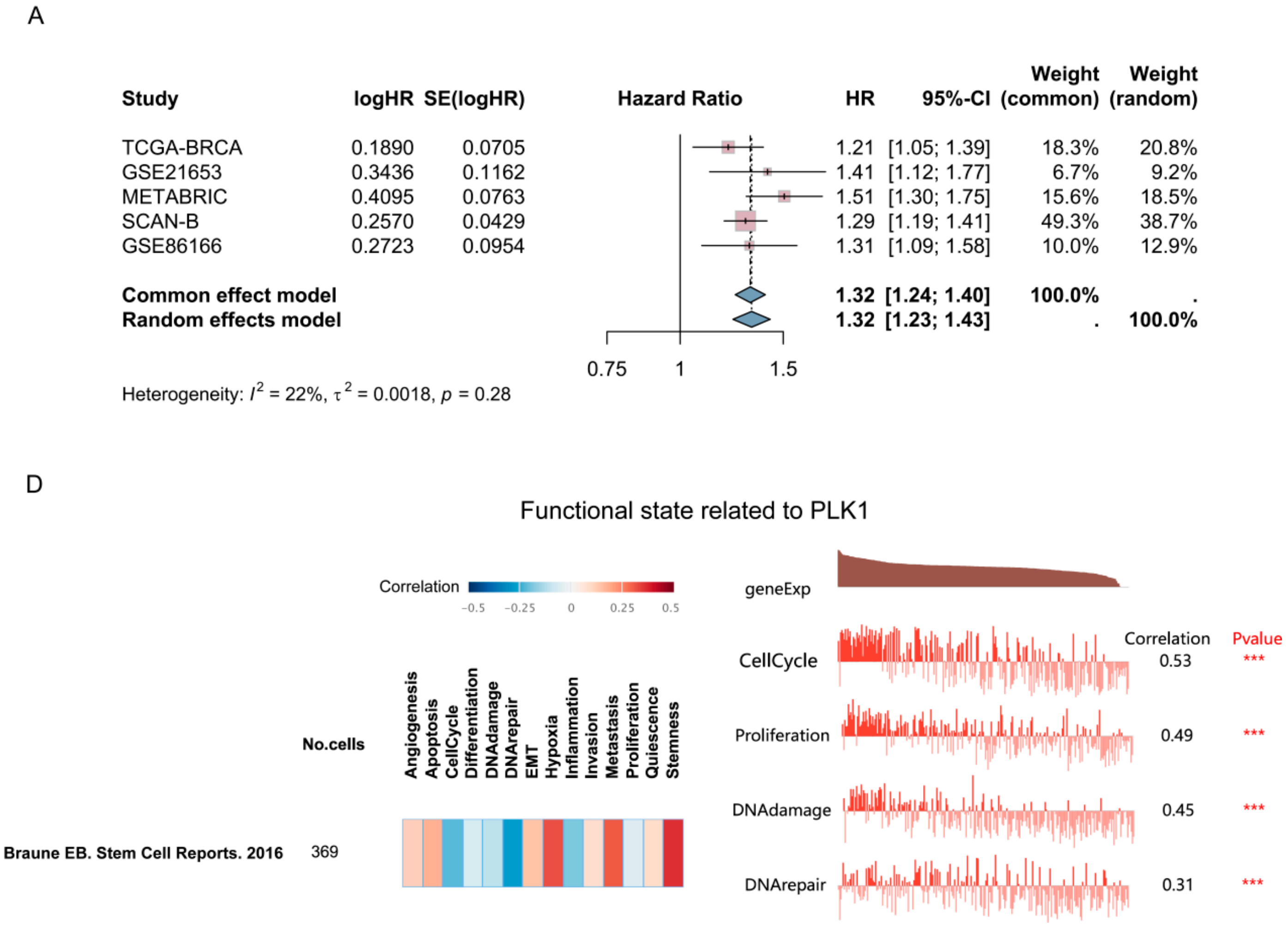

Figure S7 Comprehensive analysis of PLK1. (**A**) Forest plots of Meta-analysis on PLK1 and OS of BC. (**B**) Functional state related to PLK1.
